# Contextual influences on the role of evidence in health policy development: insights from India and Nigeria

**DOI:** 10.1186/1472-6963-14-S2-O5

**Published:** 2014-07-07

**Authors:** Tolib Mirzoev, Mahua Das, Bassey Ebenso, Bindiya Rawat, Nkoli Uguru, Giuliano Russo, Roger Bymolt, Reinhard Huss

**Affiliations:** 1 University of Leeds, Leeds, UK; 2 Association for Stimulating Know How, New Delhi, India; 3 College of Medicine, University of Nigeria Enugu Campus, Enugu, Nigeria; 4 Instituto de Higiene e Medicina Tropical, Lisbon, Portugal; 5 Royal Tropical Institute, Amsterdam, The Netherlands

## Background

The context is a complex and important influence on decision-making, affecting degree of responsiveness and people-centred health systems. Although theoretical frameworks to understand context are available, limited empirical research exists exploring contextual influences on evidence-informed health policymaking. This presentation compares contextual influences on the role of evidence in health policy development within two large countries within their continents: India and Nigeria.

## Materials and methods

In each country, the contextual influences on the development of three specific health policies were explored. The study was guided by a conceptual framework, developed from the literature. Context includes factors at three levels: macro (e.g. political and resource environment), meso (e.g. organisation’s roles and practices) and micro (e.g. individual values and preferences). Data was collected using 72 in-depth interviews with key policy actors and document reviews, and analysed using framework approach.

## Results

All policies were perceived as evidence-informed. Both formal (e.g. research) and informal (e.g. experiences) evidence was used in India; in Nigeria reliance was mostly on formal evidence. Key macro-level facilitators of evidence-informed decisions were international treaties driving reform agendas, leadership changes and political will. Key constraints included limited resources and opposition from powerful actors. At meso-level, civil society was particularly influential in India; whereas international agencies had greater role in policy decisions, including evidence use, in Nigeria. At micro-level, individuals had different understandings of what constitutes ‘robust’ evidence for policymaking, shaping their evidence preferences and decision-making practices.

## Conclusions

Understanding context is essential in ensuring responsiveness of policy decisions to the needs of key policy actors within people-centred systems, for example through recognising actors’ agendas and interests. Powerful civil society can catalyse greater recognition of citizens voice through communicating informal evidence, as we found in India; and influential donors can favour costly surveys, thus undermining use of evidence from government health information systems, as in Nigeria.

